# Computational evidence for nonlinear feedforward modulation of fusimotor drive to antagonistic co-contracting muscles

**DOI:** 10.1038/s41598-020-67403-w

**Published:** 2020-06-30

**Authors:** Russell L. Hardesty, Matthew T. Boots, Sergiy Yakovenko, Valeriya Gritsenko

**Affiliations:** 1 Neural Engineering and Rehabilitation Laboratory, Division of Physical Therapy, School of Medicine, West Virginia University, Morgantown, WV USA; 2 Neural Engineering Laboratory, Division of Exercise Physiology, School of Medicine, West Virginia University, Morgantown, WV USA; 3 Department of Mechanical and Aerospace Engineering, Benjamin M. Statler College of Engineering and Mineral Resources, West Virginia University, Morgantown, WV USA; 4 Rockefeller Neuroscience Institute, West Virginia University, Morgantown, WV USA

**Keywords:** Dynamical systems, Spinal cord, Reflexes, Neurophysiology, Control theory

## Abstract

The sensorimotor integration during unconstrained reaching movements in the presence of variable environmental forces remains poorly understood. The objective of this study was to quantify how much the primary afferent activity of muscle spindles can contribute to shaping muscle coactivation patterns during reaching movements with complex dynamics. To achieve this objective, we designed a virtual reality task that guided healthy human participants through a set of planar reaching movements with controlled kinematic and dynamic conditions that were accompanied by variable muscle co-contraction. Next, we approximated the Ia afferent activity using a phenomenological model of the muscle spindle and muscle lengths derived from a musculoskeletal model. The parameters of the spindle model were altered systematically to evaluate the effect of fusimotor drive on the shape of the temporal profile of afferent activity during movement. The experimental and simulated data were analyzed with hierarchical clustering. We found that the pattern of co-activation of agonistic and antagonistic muscles changed based on whether passive forces in each movement played assistive or resistive roles in limb dynamics. The reaching task with assistive limb dynamics was associated with the most muscle co-contraction. In contrast, the simulated Ia afferent profiles were not changing between tasks and they were largely reciprocal with homonymous muscle activity. Simulated physiological changes to the fusimotor drive were not sufficient to reproduce muscle co-contraction. These results largely rule out the static set and α-γ coactivation as the main types of fusimotor drive that transform the monosynaptic Ia afferent feedback into task-dependent co-contraction of antagonistic muscles. We speculate that another type of nonlinear transformation of Ia afferent signals that is independent of signals modulating the activity of α motoneurons is required for Ia afferent-based co-contraction. This transformation could either be applied through a complex nonlinear profile of fusimotor drive that is not yet experimentally observed or through presynaptic inhibition.

## Introduction

Movement is the product of interactions between neural signals and the musculoskeletal dynamics that depends on limb anatomy^1–4^. The motor control problem is then solved within a system with coupled neural and mechanical dynamical elements^5–7^. Therefore, the relationship between neural signals driving muscle contraction and the resulting motion is nonlinear. Muscle contractions generate forces that sum into active moments defined by the agonistic or antagonistic relationships between the muscle’s moment arms around a given axis of rotation of the joint. The components of these forces that sum to zero moment, such as forces produced by balanced co-contraction of antagonistic muscles, define joint stiffness and viscosity. The remaining unbalanced moments are often termed muscle torques; they produce motion. In this bottom-up reasoning, muscle contractions represent the output of the central nervous system (CNS) that also reflects the mechanical properties of the limb being moved by these muscles. For example, muscle torques derived from motion capture share a large amount of variance with muscle activity profiles during reaching movements in certain directions^8^, while in other directions co-contraction defines the muscle activity profiles more than muscle torques. Joint stiffness is the product of co-contraction of antagonistic muscles. However, without the knowledge of the moment arms and motor unit recruitment of these muscles, it is often difficult to estimate experimentally joint stiffness from surface electromyography. Some studies estimate co-contraction using “wasted contraction”, i.e. the minimal value of estimated muscle recruitment between antagonistic muscles^9–11^. Other studies measure joint stiffness more directly with perturbations^12–14^. These studies found that both co-contraction and stiffness change with task demands, so that the co-contraction increases in the novel, precise, or demanding tasks and the resulting stiffness depends on limb dynamics. The joint and, possibly, whole arm stiffness is thought to be a controlled parameter in the CNS ensuring movement stability^15,16^. An important question in motor control is how the co-contraction of antagonistic muscles that modulate stiffness with stability is produced.

It is well established that at the lowest level of the CNS, the primary afferents (Ia) from muscle spindles can increase the activity of the homonymous muscle and its agonists, thus increasing their stiffness, through the homonymous monosynaptic reflex^17^. It is also well established that the same Ia feedback through an interneuron can inhibit the activity of the antagonistic muscle, contributing to the reciprocal muscle activation during locomotion^18,19^. The contribution of these pathways to muscle activation can be modulated via the activity of dynamic and static γ motoneurons that change the profile of activity of the Ia afferent^20^. During movement, the dynamic fusimotor action changes mainly the velocity sensitivity of the Ia afferents, while the static fusimotor action changes mainly the length sensitivity of the Ia afferents^21,22^. How exactly the activity of γ motoneurons changes during reaching movements in humans is unknown (for the reviews of afferent recording studies see^22,23^). However, the effect the fusimotor drive has on shaping the muscle spindle output can be broadly classified based on whether the fusimotor drive is constant or changing during movement. The former is defined as the static set, where γ motoneuron activity remains constant during a given movement, but its level changes between different movement types adjusting muscle spindle sensitivity to the anticipated demands of the task^24,25^. Alternatively, the Ia feedback could be coupled to the ongoing motor activity via α-γ coactivation, where the sensitivity of muscle spindles is maintained during muscle shortening by coupling the activity of γ motoneurons to the activity of α motoneurons^26,27^. The fusimotor drive provided by β motoneurons, which innervate both extrafusal and intrafusal muscle fibers^28^, can also modulate Ia afferent activity. However, in our correlative study, the effect of β motoneurons is indistinguishable from the effect of α-γ coactivation. Given such complex and flexible Ia feedback that could be transmitted through the mono- and disynaptic pathways, the role that it plays in co-contraction and ultimately limb stiffness is unknown. It has been suggested the co-contraction of antagonistic muscles can be modulated by descending signals through the concurrent fusimotor drive, e.g. C command in lambda-model^29^. A pathological change in the strength of the monosynaptic connection of Ia afferents to α motoneurons is also implicated in spasticity, a condition that is characterized by abnormal co-contraction of antagonistic muscles^30^. The question arises whether the common fusimotor drive to muscle spindles in antagonists can contribute significantly to their co-contraction through the monosynaptic Ia feedback under normal conditions, such as during reaching. Answering this question will help constrain the space of possible solutions for descending neural control signals.

The current methods of directly observing primary afferent firing in humans, such as microneurography, are limited in the number of observable signals and the types of behaviors these observations can be made under. In presence of these limitations, the experimentally validated models of primary afferents^31^ and the musculoskeletal anatomy of the arm^32^ used together can provide unique insight into the transformation through the motoneuron pool. Muscle spindle models have been used to gain valuable insight into the contribution of afferent feedback during healthy and injured locomotion^31,33,34^ The novel approach used here enables a holistic computational estimation of the Ia afferent activity from multiple muscles during reaching movements in humans. Here, we used the model of muscle spindle with the two types of fusimotor drive, static set and α-γ coactivation, to address the question of Ia afferent contribution to the co-contraction of multiple muscles during reaching movements. The movements were selected based on the roles of passive forces, assistive or resistive, during reaching that were expected to be accompanied by different patterns of muscle co-contraction. We then used a mathematical model of Ia afferent with two parameters that define the sensitivity of muscle spindle to muscle length and velocity changes^31^. We changed these parameters across tasks according to the experimental observations that informed the two types of fusimotor drive. We took advantage of the linearizing properties of the motoneuron pool in transforming synaptic drive into neural command to the muscles^35^. We used electromyography (EMG) to estimate the ensemble activity of the motoneuron pool^35–37^. During reaching without heavy objects, the maximal EMG in arm muscles is estimated to be low, 5–10% of maximal voluntary contraction^38,39^. At that range, the relationship between EMG and the recruitment of motoneuron pool is largely linear^36^. This justified employing a hierarchical clustering analysis to quantify the linear relationships between time-varying muscle activity (EMG), including co-contraction, and simulated Ia afferent activity. We expected that the activity of co-contracting antagonistic muscles will positively correlate with the activity of their Ia afferents shaped by static set and α-γ coactivation, which would be evident from observing these signals in the same clusters.

## Materials and methods

### Experimental design and human participants

We recruited 9 healthy adults (5 males, 4 females; age, 24.3 ± 1.8 years; weight, 76.3 ± 14.5 kg) to perform reaching movements to visual targets in a virtual reality (VR, Oculus Rift, developer kit 2). All procedures were approved by the West Virginia University Institutional Review Board (IRB). All methods were performed in accordance with the IRB guidelines and regulations; informed consent was obtained from all individuals prior to their participation in the study. All data analysis and simulations were performed in Matlab (MathWorks, RRID:SCR_001622).

**Figure 1:**
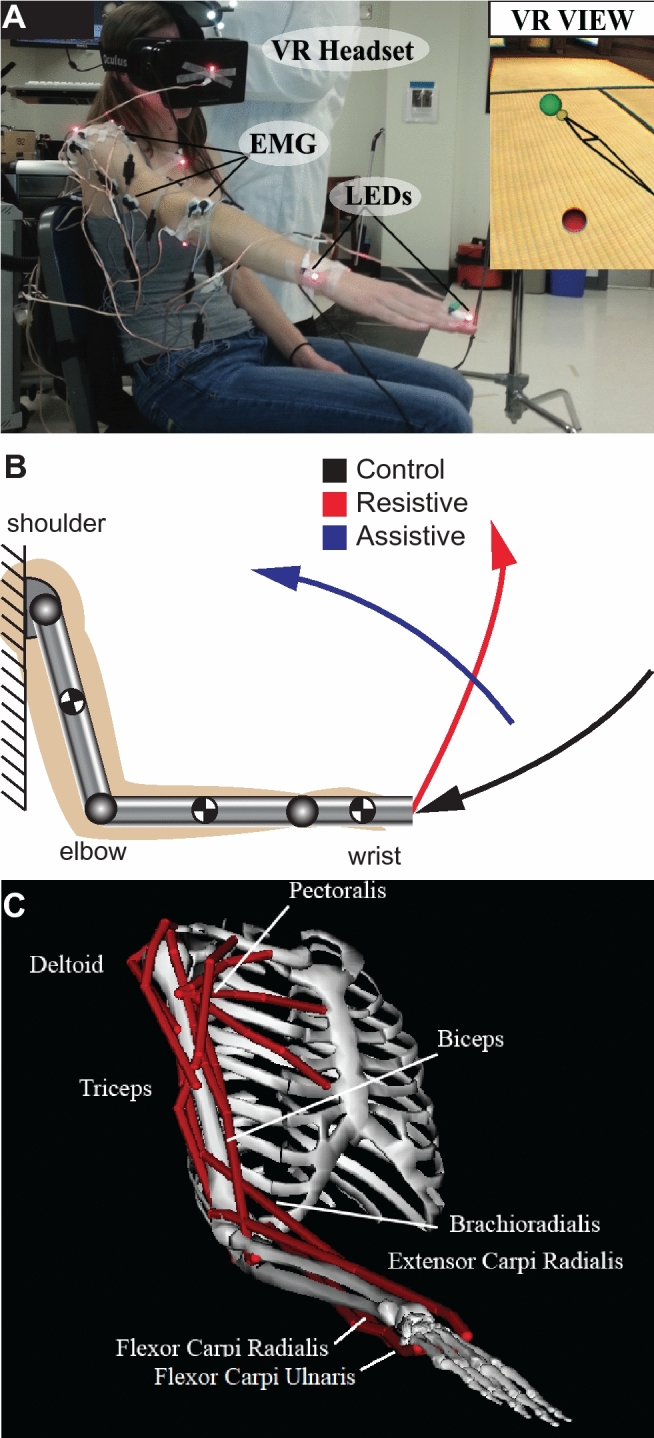
Illustrations of the experimental setup and arm models. Oculus DK1 is shown, but all data collection occurred with Oculus DK2 headset. (**A**) Annotated photo of the setup; insert shows participant's monocular view. Reaching target is in green, the origin target is in red. Yellow sphere shows the location of individual's fingertip and the black lines outline the major arm segments for visual feedback of arm location in VR. (**B**) Colored lines show the fingertip trajectories of each of the three tasks. Arrows indicate the direction of motion toward the reaching target. The grey blocks show the locations and orientations of local coordinate systems used to obtain joint torques from motion capture. Circles with black and white quarters indicate the locations of the centers of mass and the orientations of local coordinate systems. (**C**) Illustration of the OpenSim model used to derive muscle lengths for the calculations of Ia afferent discharge. Red lines show the anatomical paths of each muscle from which EMG signals were recorded during experiments.

Participants performed three reaching tasks in VR (Fig. 1A). Pairs of visual targets defined the starting and goal target locations for each task (Fig. 1B). The virtual environment provided two distinct advantages: (1) it allowed target locations to be quickly calculated and scaled based upon an individual participant’s proportions and (2) it provided visual guidance to constrain movement trajectories without physically interacting with the participant, i.e. altering limb dynamics. To minimize inter-subject variability in angular kinematics, the locations of virtual targets were derived using planar trigonometry based on the lengths of individual's arm and forearm segments and displayed relative to the subject’s shoulder location in VR. This resulted in the same shoulder, elbow, and wrist angles at the start and end of each movement across participants. The pairs of starting and goal visual targets were shown in a random sequence to minimize bias. The cue to move was the change of target color from red to green. Trunk motion was restricted with straps, wrist was instructed to be kept at neutral palm down (Fig. 1A). Each task was repeated 24 times. At the beginning of each 60-trial block, the virtual target positions were re-calibrated to the participant’s shoulder location.

The tasks were based on planar pointing movements selected for their diverse dynamical contexts. The Control movement (Fig. 1B, black) was largely passive with the arm being lowered with gravity. The Resistive movement (Fig. 1B, red) was accompanied by increasing gravitational load at the shoulder and resistive interaction torques between the shoulder and elbow^40^. Finally, the Assistive movement (Fig. 1B, blue) was accompanied by decreasing gravitational load at the shoulder and assistive interaction torques between the shoulder and elbow. The dynamical contexts were identified based on inverse simulations in Simulink (RRID:SCR_014744) with a mechanical planar model of the arm^8^ that predicted shoulder and elbow torques for a given linear trajectory between an arbitrary set of starting and ending postures.

During the performance of each task, we recorded the kinematics of the shoulder, elbow, and wrist joints and electromyography (EMG) of 12 muscles that span those joints. The recorded muscles were the anterior and posterior deltoids (AD and PD, respectively), pectoralis major (Pec), teres major (TM), biceps brachii long and short heads (BicL and BicS, respectively), triceps brachii lateral and long heads (TriLa and TriLo, respectively), brachioradialis (Br), extensor carpi radialis (ECR), flexor carpi radialis (FCR), and flexor carpi ulnaris (FCU). These muscle abbreviations are used consistently throughout the manuscript and figures. Motion capture data were recorded at 480 Hz using an Impulse system (PhaseSpace), and EMG signals were recorded at 2000 Hz with an MA400-28 system (MotionLab Systems). Nine LED markers were placed on bony landmarks of the arm and trunk (Cervical Vertebrae 7, Xiphoid Process, Sternoclavicular Joint, Acromial Edge, Acromioclavicular Joint, Lateral Olecranon Process, Radioulnar Joint, Styloid Process, and the Distal Phalanges Head). The start and end of each movement was defined by finding a local maximum in the 3rd derivative of the vector distance profile of the wrist and elbow LED markers. The motion capture data were used to derive joint angles by fitting local coordinate systems into the markers defining each major segment and deriving Euler angles between them using linear algebra^41^. The EMG was processed consistent with SENIAM recommendations, it was high-pass filtered at 10 Hz, rectified, and low-pass filtered at 20 Hz. The resulting EMG profiles were time-normalized between onset and offset of each movement, averaged per task, and amplitude-normalized to the maximum across all tasks per participant. Co-contraction was calculated as “wasted contraction”^9–11^ between normalized EMG profiles of pairs of antagonists defined as follows, AD-PD, Pec-TM, BicL-TriLo, BicS-TriLa, Br-TriLa, FCR-ECR, and FCU-ECR.

### Primary afferent model

To estimate the sensory contribution from muscle spindles during movement, we used Prochazka’s model of primary afferent discharge^42^, which offers a clear parametrization of static and dynamic responses. The spindle model relates afferent firing rate (*Ia*) to the time-varying muscle length (*l*) and its rate of change (*v*) as follows:

1 $$Ia(v,l)=A{v}^{0.5}+Bl+C$$

where the constant parameters. In Eq. (1) (*A* = 65, *B* = 200, and *C* = 10) were validated empirically to reflect human microneurography data^43^.

The changes of musculotendon length during movement were calculated in OpenSim (RRID:SCR_002683) using a modified musculoskeletal model of the human arm^32^ (Fig. 1C). This model was adjusted for each individual using segment lengths to scale model’s segments and move proportionally the origin and insertion of each simulated muscle. Muscle lengths were simulated by driving the adjusted model with the mean angular trajectories for each task and participant. This resulted in temporal profiles of muscle length ($l$) in units of meters and its derivative ($v$) in units of meters per second for each movement per participant. Muscle length profiles used in the Ia model were converted to the rest-length units based on the minimal and maximal muscle lengths observed across all the possible postures of the OpenSim model in Gritsenko et al.^1^. The rest length was defined as half the length between the maximal and minimal muscle length values^33^. The muscle shortening/lengthening velocity profiles used in the Ia model were converted to the rest length per second units. The parameter space of *A* and *B* variations was explored in the context of variable fusimotor drive. To simulate a change in the dynamic fusimotor drive, we varied the velocity coefficient *A*; to simulate a change in the static fusimotor drive, we varied the length coefficient *B*. The following parameter ranges were explored: *A* ∈ [33 200] and *B* ∈ [50 400], which resulted in 4 models of static set referred to below as follows: V33-L50, V33-L400, V200-L50, V200-L400, where V stands for velocity coefficient and L stands for muscle length coefficient. This also served as a sensitivity analysis of the two parameters of the Ia model.

Separately, we approximated α-γ coactivation that affects both the dynamic and static fusimotor drive using EMG profiles which transformed Eq. (1) as follows:

2 $$Ia(a,v,l)=a\cdot 65{v}^{0.5}+a\cdot 200l+10$$

where *a* is the normalized mean EMG profile for a given task and participant. The model in Eq. (2) is referred to below as EMG-coupled Ia model.

The time-varying primary afferent profiles calculated with Eqs. (1) and (2) are referred to below as Ia profiles. For the regression analysis described below, the Ia profiles were amplitude-normalized to the maximum across all tasks per participant to obtain unitless values.

### Verifying task dynamics

**Figure 2:**
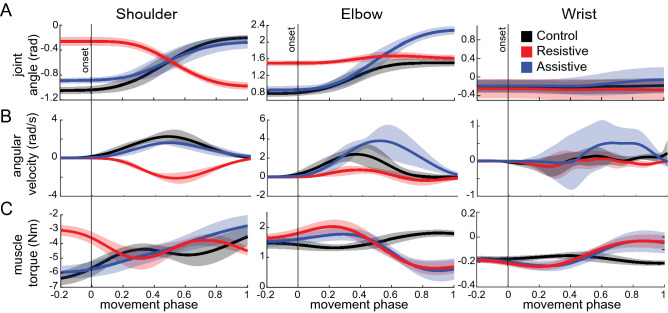
Signals calculated from motion capture. Thick lines show normalized mean trajectories for each movement across all participants, shaded areas show standard deviations across participants. Movement phase represents the normalized duration of each movement with 0 indicating the start of movement (vertical onset line) and 1 indicating the end of movement. (**A**) Joint angles; (**B**) joint angular velocity; (**C**) muscle torques.

A mechanical model of a human upper-limb^8^ was used to compute joint torques from joint angles inferred from motion capture. The mechanical model described above was expanded to comprise three segments and five degrees of freedom, including the shoulder (flexion/extension, abduction/adduction, internal/external rotation), elbow (flexion/extension), and wrist (flexion/extension). The height and weight of each individual were used with anthropometric tables^44^ to estimate the lengths and cylindrical inertias of the arm, forearm, and hand segments (Fig. 1B). To calculate active torques that result from muscle action, mean angular trajectories from each individual and task were used to drive the subject-specific model in inverse dynamic simulations (Fig. 2). The motion defined by our tasks was in the vertical plane. Participants showed minimal out-of-plane motion as measured by angular trajectories about shoulder abduction/adduction and internal/external rotation degrees of freedom. Therefore, only muscle torques about the shoulder flection/extension degree of freedom was included in the analysis described below.

To describe the limb dynamics of each task, the three muscle torques about shoulder, elbow, and wrist were then used to calculate the following parameters. (1) The postural torque change was calculated as the difference between muscle torques averaged over 100 ms prior to onset and following the offset of movement. These postural torques are produced to maintain the arm in starting and final postures against the force of gravity. (2) The peak torque change in acceleration phase was calculated as the maximal change in torque between the start of movement to its halfway point. The first half of movement was used to ascertain the amount of muscle force that is required to start the motion, which is thought to represent largely feedforward activation. (3) The mechanical muscle work was calculated by integrating a product between muscle torques and angular velocity as described in^44^. When the direction of action matches between muscle torque and angular velocity, as indicated by the same sign (both positive or both negative), the mechanical muscle work is positive. This means that agonist muscle contractions about the corresponding degree of freedom are concentric and they are actively producing the motion. When the direction of action is opposite between muscle torque and angular velocity, as indicated by opposite signs, the mechanical muscle work is negative. This means that agonist muscle contractions about the corresponding degree of freedom are eccentric and the motion is produced by passive torques, such as gravity, interaction torques, etc. In our tasks, the wrist joint does not move, therefore the mechanical muscle work about the wrist is zero. This means that the muscle torque about the wrist reflects the isometric contraction of wrist and hand muscles that is required to stabilize the joint at a constant angle.

### Analysis

To quantify the common and distinct features in EMG and Ia profiles and to compare them to features obtained from muscle lengths we used hierarchical clustering. The relationships between the normalized averaged EMG and Ia profiles were characterized by a matrix of Pearson’s correlation coefficients (*r*). To reduce the probability of Type I errors, the α for determining the significance of *r* values was adjusted using the two-stage Benjamini, Krieger, and Yekutieli procedure for controlling false detection rate^45^. The correlation matrix was then transformed into the heterogeneous variance explained (HVE) as follows:

3 $$HVE=\left\{\begin{array}{l}1-{r}^{2},\left|r>0, p<\alpha \right.\\ 1+{r}^{2},\left|r<0, p<\alpha \right.\\ 1,\left|p\ge \alpha \right.\end{array} \right.$$

The HVE from Eq. (3) transforms the large positive *r* values that are characteristic of agonistic relationships into short distances close to 0 and the large negative *r* values corresponding to antagonistic relationships into long distances close to 2. To identify synergistic relationships between EMG and Ia, we applied hierarchical clustering to an unbiased HVE distance matrix using the linkage function with an unweighted average distance method^1^. The goodness of fit of the clustering model was assessed using the cophenetic correlation coefficient, which quantified how faithfully the hierarchical cluster tree represented the dissimilarities among observations. The magnitude of this value should be very close to 1 for a high-quality solution. As a result of this analysis, the strongly and positively correlated signals will be labeled belonging to the same cluster, and we will be able to assess the degree of similarity between these clusters based on the strength of the positive and negative correlations between them. This approach is advantageous in examining the correlation structure while still distinguishing between positive and negative correlations. Hierarchal cluster analysis has captured the relationship between EMG and Ia signals with high precision, as evidenced by high cophenetic coefficient of 0.81 ± 0.044, mean and standard deviation across participants.

Clusters were compared using the Fowlkes–Mallows index (*B*_*k*_) to assess cluster similarity between separate hierarchical cluster trees^46^. The Fowlkes–Mallows index represents a normalized number of common elements between clusters from different trees at the same cluster height. For example, *B*_2_ indicates that the hierarchical trees were compared at the height, where only 2 clusters occur. Here, we explored *k* = [2,…, *n*], where *n* is half the number of signals being included in the hierarchical clustering. Thus, for two cluster trees with arbitrarily numbered clusters *i* = 1,…, *k* and *j* = 1,…, *k* we can use the number of objects between the *i*th cluster of one tree and *j*th cluster of the other tree (*m*_*ij*_) to calculate the index as follows:

4 $${B}_{k}=\frac{{T}_{k}}{\sqrt{{P}_{k}\cdot {Q}_{k}}},$$

where

5 $${T}_{k}=\sum_{i=1}^{k}\sum_{j=1}^{k}{m}_{ij}^{2}-n,$$

6 $${P}_{k}=\sum_{i=1}^{k}{\left(\sum_{j=1}^{k}{m}_{ij}\right)}^{2}-n,$$

7 $${Q}_{k}=\sum_{j=1}^{k}{\left(\sum_{i=1}^{k}{m}_{ij}\right)}^{2}-n,$$

At each cluster division, the index is calculated such that 0 ≤ *B*_*k*_* ≤ 1,* where *B*_*k*_ = 1 indicates two identical clusters and provides a means to compare the multi-muscle clustering of EMG and Ia signals. A further benefit of the Fowlkes–Mallows index is that it approaches 0 with an increasing number of data points, making it less sensitive to spurious correlations than the commonly used Rand index^46^.

In an earlier study^1^, we quantified the synergistic relationships between muscles based on their anatomy using the same musculoskeletal model of the arm used here for muscle length measurements. The muscle lengths were calculated over the whole range of physiological joint postures and analyzed using the same hierarchal clustering method described above. Here we selected a subset of muscles recorded in this study and compared the clustering structure of the muscle lengths across all postures in Gritsenko et al.^1^ study to the clustering structure of simulated Ia afferent activity. We used the muscle lengths obtained from Gritsenko et al.^1^ rather than the muscle lengths calculated for the Ia modeling, because the former was calculated over a wider range of postures than the latter. Because the muscle lengths data is part of the Eq. (1), similar clustering structure is expected between muscle lengths and Ia profiles. These muscle length data were used as one of the controls for the statistical analysis of clustering structure described below.

### Statistics

All values reported in results are means with standard deviations across participants, unless stated otherwise. The shared variance (R^2^) between clusters defined by hierarchical clustering was assessed using t-tests. The t-tests were applied to R^2^ values averaged across members of the cluster per participant per task. Individuals were assumed to represent independent samples. The combined p-values across participants included in the tables were obtained using the Fisher's combined probability test^47^. Correction for multiple testing was based on Bonferroni adjustment of alpha, the acceptable probability of making type I error^48^.

The statistical comparison of hierarchical clustering between multiple signal modalities was based on permuting the hierarchical clustering trees to estimate the chance of observing spurious correlations. The hierarchal tree for each participant each movement type and each signal modality (Ia, EMG, muscle length) was randomly permuted 1,000 times. Then the Fowlkes–Mallows index (*B*) was calculated between each of the permuted trees, which resulted in a population of *B* values that represents the distribution of noise. The distribution of experimental *B* values across tasks and individuals was compared to the corresponding noise distribution of *B* values to test the hypothesis that *B*_*experimental*_* ≠ B*_*noise*_. The p-value for each experimental B value was determined from the corresponding noise distribution for each individual using the percentile method^49^. The combined p-values across cluster subdivisions included in the supplementary tables were obtained using the Fisher's combined probability test^47^. The significant alpha was set to 0.0056 to adjust for repeating tests across 9 participants. This permutation analysis was applied to test three hypotheses. The 1st hypothesis was that the similarity between Ia and muscle-length clusters is not spurious. This is a test of the chosen statistical method. We expect to support the 1st hypothesis, because the Ia and muscle length profiles are not independent, i.e. the former is derived from the latter as described in Eq. (1). The 2nd hypothesis was that the similarity between Ia and EMG clusters is not spurious. The 3rd hypothesis was that EMG and Ia clusters change the same way between tasks. Supporting either 2nd or 3rd hypothesis means that the compared trees are similar to a greater extent than is expected by chance, and that the afferent activity clusters comprise the same muscles as the muscles that co-activate in a given task.

The statistical comparison of hierarchical clustering between tasks was based on bootstrapping the *B* values^49^. The *B* values for EMG clustering (*B*_*EMG*_) and Ia clustering (*B*_*Ia*_) calculated between tasks were resampled with replacement 1,000 times. This resulted in two distributions of 45,000 *B*_*EMG*_ and *B*_*Ia*_ values for each task pair (Control-Resistive, Control-Assistive, and Resistive-Assistive). These data were used to test the 3rd hypothesis that EMG and Ia clusters change the same way between tasks. To test this hypothesis, we calculated the difference between the two distributions of *B*_*EMG*_ and *B*_*Ia*_ values across tasks, each comprising 1,000 bootstraps per cluster number (*k* = 2,…, 6) per participant (N = 9). The p-value for each task pair was determined from the location of the 0 value in the resulting distribution of differences, which indicated no difference between cluster structures, using the percentile method^49^.

The last set of hypotheses addressed the extent to which the fusimotor drive can shape Ia afferent discharge and capture muscle co-contraction. The hypothesis for each altered Ia model was that the similarity between Ia and EMG clusters is increased by alternative fusimotor drives. To test these hypotheses, the *B* values were calculated between the hierarchical clustering of Ia and EMG profiles for each of the models with altered coefficients (V33-L50, V33-L400, V200-L50, V200-L400, and EMG-coupled). The distribution of *B* values from each of the altered model was subtracted from the corresponding *B* values based on Ia profiles from the Prochazka model. The p-value for each model with altered coefficients was determined based on the location of the 0 value in the resulting distribution of differences using the percentile method^49^.

## Results

Participants performed reaching tasks with consistent angular kinematics within the constraints defined by the VR targets. The angular excursions of each joint were similar across individuals for the three tasks (Fig. 2A), the angular velocity was most variable across participants in the Assistive task (Fig. 2B). Because the individual’s movements were not restricted, most participants moved slightly out of the sagittal plane and the experimental angular displacement differed somewhat from those defined by virtual targets (Table 1). In the Control and Assistive tasks, the virtual targets defined joint excursions that required the shoulder and elbow joints to rotate in opposite directions. This caused assistive interaction torques between these joints similar to the Assistive task in Gritsenko et al.^40^, which were associated with negative muscle work at the shoulder and positive muscle work at the elbow (Table 1). The sign of work indicates the direction of energy flow. The positive sign of work indicates concentric contractions that transfer energy from muscles to segments, while the negative sign of work indicates eccentric contractions during which the energy from external forces are overpowering the muscle action and doing the work^44^. Thus, in the Control and Assistive tasks, the shoulder motion was largely passive, and the activity of shoulder muscles was compensating for external forces due to gravity and interaction torques. In the Control task, elbow and wrist torques were the lowest across the three tasks (Fig. 2C, black lines). The Assistive task was accompanied by decreasing postural torques in all joints, low acceleration shoulder torques, but high deceleration elbow and wrist torques (Fig. 2C, blue lines; Table 1, third column). This shows that in the Assistive task most of the muscle action was to decelerate the limb accelerated primarily by the interaction torques and gravity. In contrast, in the Resistive task the joint excursions were such that required the shoulder and elbow to rotate in the same direction, causing resistive interaction torques similar to the Resistive task in^40^. Altogether, this caused the opposite pattern of shoulder torques compared to that in the Control and Assistive tasks, while maintaining the same elbow and wrist torques to that in the Assistive task (Fig. 2C). In the Resistive task, the mechanical muscle work was always positive, indicating that muscle contractions were concentric and that the motion was produced with the least reliance on passive limb dynamics and gravity.

**Table 1:** Table of task parameters.

	Control	Resistive	Assistive
**Shoulder**			
Target-defined excursion (deg)	− 60	− 45	− 40
Experimental excursion (deg)	− 48 ± 2 E	40 ± 4 F	− 35 ± 5 E
Postural torque change (Nm)	− 2.7 ± 0.3 F	1.3 ± 0.5 F	− 3.2 ± 0.5 F
Peak torque change in acceleration phase (Nm/s)	8.3 ± 3.6 E	9.0 ± 3.1 F	4.5 ± 1.8 E
Mechanical muscle work (J)	− 3.80 ± 0.39	3.05 ± 0.42	− 2.60 ± 0.23
**Elbow**			
Target-defined excursion (deg)	60	10	100
Experimental excursion (deg)	42 ± 4 F	7 ± 3 F	81 ± 8 F
Postural torque change (Nm)	0.3 ± 0.2 F	− 1.0 ± 0.2 F	− 0.9 ± 0.4 F
Peak torque change in acceleration phase (Nm/s)	1.8 ± 0.5 F	5.2 ± 2.3 E	5.3 ± 3.4 E
Mechanical muscle work (J)	0.99 ± 0.20	0.28 ± 0.06	1.93 ± 0.24
**Wrist**			
Target-defined excursion (deg)	0	0	0
Experimental excursion (deg)	0.7 ± 4.3 N	1.4 ± 3.7 N	7.5 ± 13.3 E
Postural torque change (Nm)	0.0 ± 0.03 N	− 0.15 ± 0.05 F	− 0.14 ± 0.10 F
Peak torque change in acceleration phase (Nm/s)	0.3 ± 0.2 E	0.8 ± 0.3 F	0.9 ± 0.6 F
Mechanical muscle work (J)	− 0.00 ± 0.01	0.00 ± 0.01	− 0.01 ± 0.03

All joint angles are around flexion/extension axes of rotation for the three major joints included in the analysis. The sign of angles and torques indicates the direction of change of the corresponding measure, positive for increase and negative for decrease. Experimental values are averages with standard deviations across participants. Target-defined excursion is different from the experimental excursion due to out-of-plane arm motion. F indicates flexion direction of action; E indicates extension direction of action; N indicates no change. Negative values of mechanical muscle work imply work done by external forces, e.g. gravity and reaction from distal segments in the mechanical chain.

**Figure 3:**
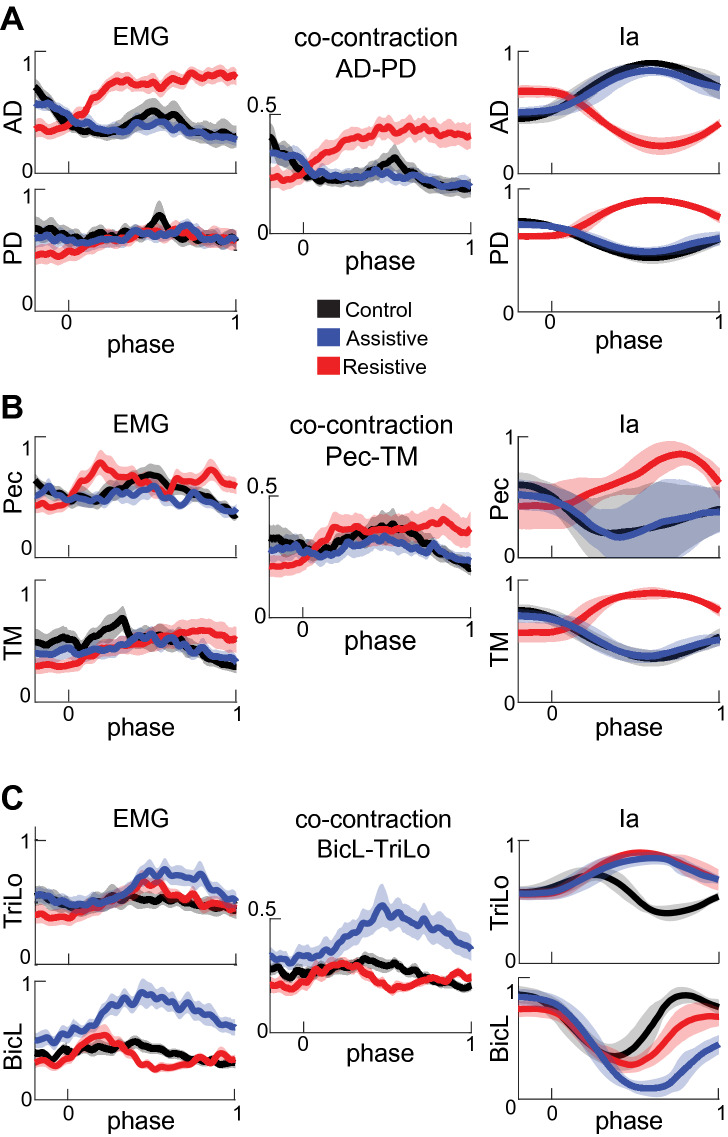
Normalized EMG, co-contraction, and Ia profiles for muscles spanning the shoulder. Thick lines show averages for each movement across all participants. Shaded areas show the standard error of the mean across participants for EMG and co-contraction signals and standard deviation across participants for Ia signals. Movement phase represents normalized duration of each movement as in Fig. 2. Signals are subdivided into antagonist pairs AD-PD (**A**), Pec-TM (**B**), and BicL-TriLo (**C**).

**Figure 4:**
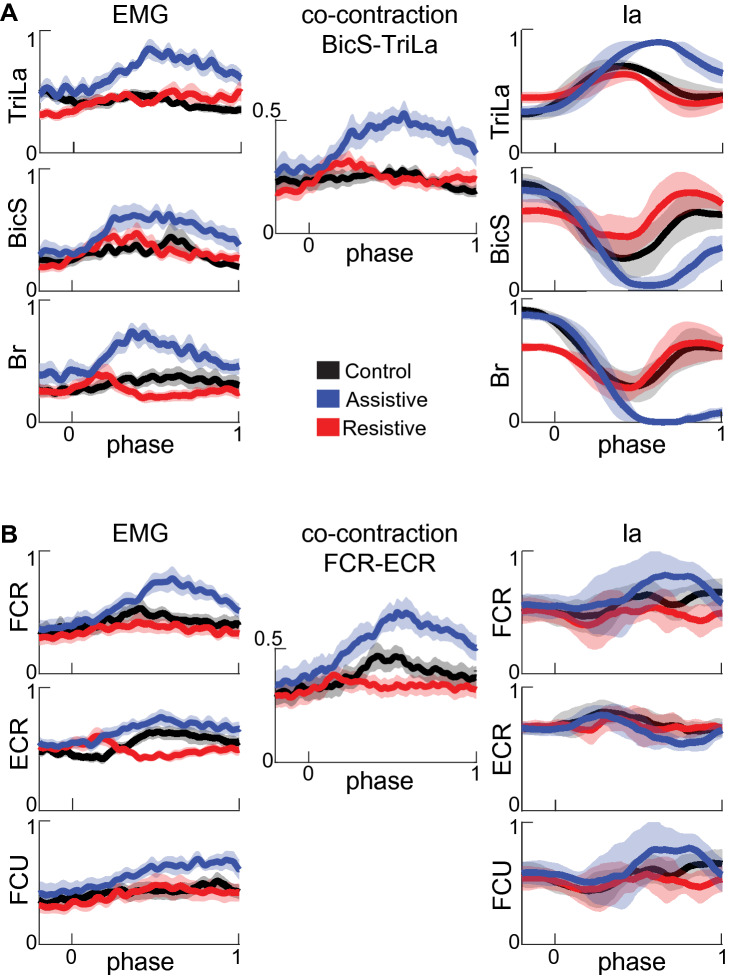
Normalized EMG, co-contraction, and Ia profiles for muscles spanning the elbow and wrist. The formatting of plots is as in Fig. 3. Signals are subdivided into antagonist pairs Br, BicS-TriLa (**A**) and FCU, FCR-ECR (**B**).

**Figure 5:**
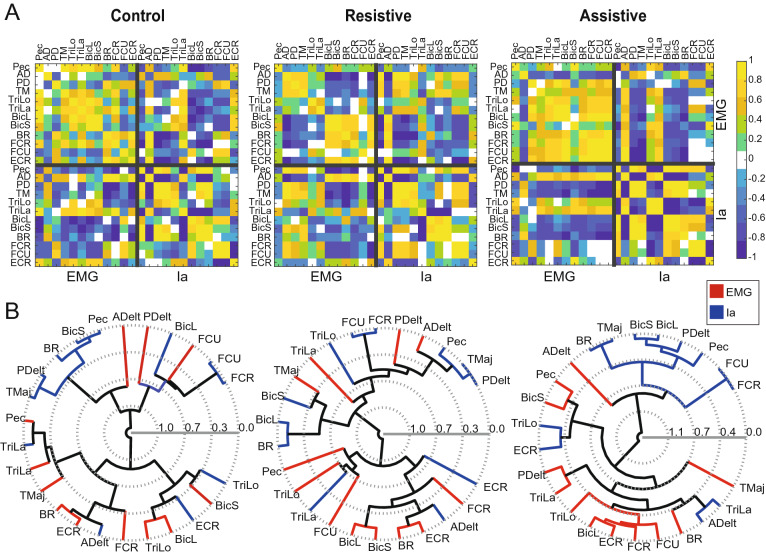
The relationships between EMG and Ia profiles per task in a representative individual. (**A**) The correlation matrix between normalized EMG and Ia profiles in one participant. (**B**) The hierarchical clustering of the correlation matrix in (**A**). Lines represent the strength of the relationship between each cluster at different cluster subdivisions.

The EMG and Ia profiles varied between tasks, but in a different manner from each other (Figs. 3, 4). The EMG profiles showed variable levels of co-contraction that changed between tasks and joints. In the Assistive task, in which passive forces assist at the shoulder, the EMG profiles of multiple muscles that span the elbow and wrist joints were ramping up during movement (Figs. 3C, 4, blue lines). The co-contraction between pairs of antagonists represented by shared variance between their normalized EMG profiles was the largest in the Assistive task (Supplementary Table S1). Hierarchal cluster analysis has shown that in the Assistive task multiple agonists and antagonists spanning the elbow and wrist (TriLa/TriLo/BicL/BicS/Br/FCR/FCU/ECR) comprised a single EMG cluster, i.e. all these muscles co-activated in this task (Fig. 5 Assistive). In the Resistive task with the least reliance on passive dynamics, co-contraction was high in AD-PD (Fig. 3A) and low in elbow and wrist muscles (Figs. 3C, 4; Supplementary Table S1). Hierarchal cluster analysis has shown that in the Resistive task, muscle coactivation was present in two different smaller clusters (Br/FCR/FCU/ECR and TM/AD/PD/TriLa; Fig. 5 Resistive), while in the Control task muscle coactivation was present in even smaller clusters (Br/FCR/FCU/ECR, TriLo/TriLa, BicS/BicL, and Pec/TM; Fig. 5 Control). Overall this analysis shows that the muscle groups defined by shared variance across EMG profiles are task-dependent and that they consist of both agonistic and antagonistic muscles.

**Figure 6:**
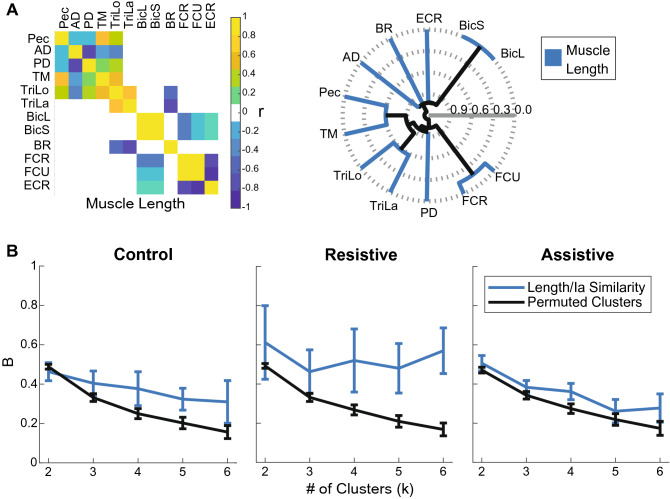
Comparison between muscle length and Ia clustering. (**A**) The relationships between muscle length profiles per task in a default “average subject” model used in^1^. The plots are formatted as in Fig. 5. (**B**) Fowkles–Mallow Index (B) for the comparison between muscle length and Ia cluster assignments (blue) and for the comparison between muscle length and permuted Ia cluster assignments representing random match (black) at different cluster subdivisions. Error bars show pooled standard deviation across participants.

In contrast to EMG profiles, the Ia profiles largely reflected the kinematic differences between tasks. For example, the Ia profiles of muscles spanning only the shoulder reversed in the Resistive task, in which the direction of shoulder excursion reversed relative to Control and Assistive tasks (Figs. 2, 3A, B); the Ia profiles of muscles spanning the elbow largely followed the profiles of elbow excursions (Figs. 2, 4). The amount of shared variance between Ia profiles from antagonistic muscles did not change between tasks and the correlations were primarily negative, except for Pec-TM (Supplementary Table S2). The negative correlations between Ia profiles of antagonists are consistent with the reciprocal actions of antagonistic muscles. This also suggests that Pec and TM are not acting as antagonists in the selected movements. The Ia profiles of smaller groups of mainly agonistic muscles, such as Pec/PD/TM, TriLo/TriLa, BicS/BicL/Br, and FCR/FCU, were positively correlated (Fig. 5A, right bottom corners of correlation matrices). Hierarchal clustering analysis comparing Ia profiles and muscle length from Gritsenko et al.^1^ showed that the Ia clusters were significantly more similar than expected by chance to muscle length clusters for all tasks at most cluster subdivisions (Fig. 6, Supplementary Table S3). Thus, we have supported the 1st hypothesis as expected. The significant similarity index at multiple cluster subdivisions confirms that the same muscles that shorten or lengthen together also have similar Ia feedback across multiple postures or movements. Overall, this analysis suggests that the Ia afferents in synergistic muscles signal similar information related to the kinematics of reaching.

**Figure 7:**
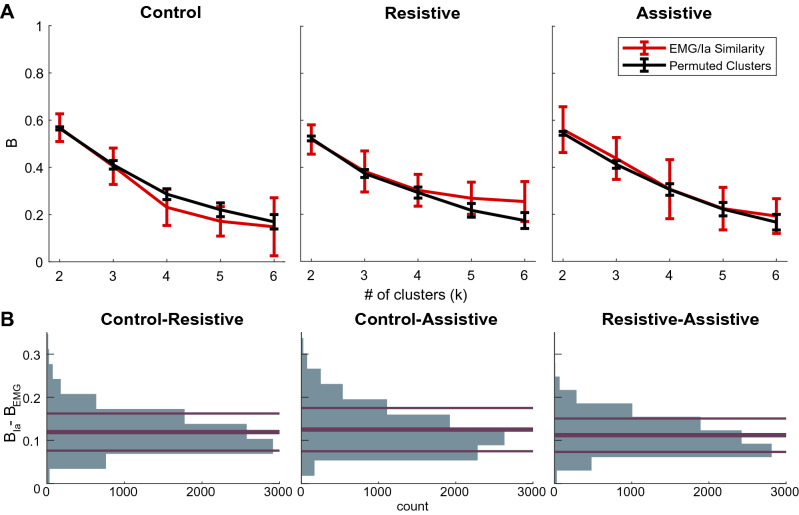
The consistency of hierarchical clustering between EMG and Ia within and across movements. (**A**) Fowkles–Mallow Index (B) for the comparison between EMG and Ia cluster assignments (red) and for the comparison between EMG and permuted Ia cluster assignments representing random match (black) at different cluster subdivisions. Error bars show pooled standard deviation across participants. (**B**) Histograms of the differences in B_Ia_ and B_EMG_ between tasks across bootstrapped hierarchal cluster trees. The abscissa indicates no difference in B values between tasks; the thick line is at the mean of the distribution; thin lines show standard deviations of the distributions.

To address the question of Ia afferent contribution to muscle co-contraction, we compared the time varying normalized Ia and EMG profiles in each task (Fig. 5A, right top corners of correlation matrices). The shared variance between Ia and EMG profiles from homonymous muscles was variable between muscles and tasks in both the strength and sign of the correlation (Supplementary Table S4). Pectoralis and biceps muscles showed the largest negative correlations, while the triceps muscle showed the largest positive correlations. This indicates that Ia feedback can both potentiate and inhibit the activity of its homonymous muscle in different tasks, even at the same level of static set across tasks represented by unchanging coefficients A and B from Eq. (1). The direct contribution of Ia afferents to muscle co-contraction can be quantified with hierarchal cluster analysis that groups positively correlated EMG profiles of co-contracting muscles and their Ia profiles into the same clusters. This predicts that, for example, the two clusters of co-activating muscles in the Resistive task should also contain the Ia profiles from the same muscles so that the Fowlkes-Mallows similarity index (*B*) between EMG and Ia clusters would lie outside the noise distribution. However, the distribution of similarity indices between EMG and Ia clusters was indistinguishable from noise at most cluster subdivisions in all tasks (Fig. 7A, Supplementary Table S5). This result did not support the 2nd hypothesis, indicating that the similarity between Ia and EMG clusters is spurious. Further analysis comparing directly the co-contraction profiles from antagonists to the Ia profiles from their host muscles further supported the lack of similarity in profiles. It showed that the Ia profiles from antagonistic muscle pairs were correlated reciprocally, either positively or negatively but never both positively, with the corresponding co-contraction profile (Supplementary Table S6). This indicates that muscle co-contraction can be potentiated by monosynaptic Ia feedback from one of the antagonistic muscles, but not both.

To contribute meaningfully to co-contraction, the Ia profiles from co-contracting muscles need to change between tasks the same way as EMG profiles of these muscles change between tasks. Therefore, we compared cluster structure between tasks. We observed that the similarity of Ia clusters (*B*_*Ia*_) between tasks was higher than the similarity of EMG clusters (*B*_*EMG*_) between tasks (Fig. 7B; p < 0.001 for all task comparisons). This shows that Ia clusters were more consistent between tasks than EMG clusters were (Fig. 6B). Therefore, the 3rd hypothesis that the EMG and Ia clusters change the same way between tasks was not supported. Instead, this result supports the conclusion above that Ia clusters contain information related to the kinematics of reaching, which changed less between our tasks than limb dynamics and co-contraction did.

Lastly, we evaluated to what extent the fusimotor drive could alter Ia signal profiles to capture muscle co-contraction. To achieve this, we manipulated Ia model coefficients to simulate alternative fusimotor inputs, such as static set and α-γ coactivation. The models of different static sets with large coefficients produced maximal firing rates that were above those reported for human large fiber afferents (Human afferents from^43^: 40 imp/s; simulated afferents from Pec: 174 ± 49 imp/s; AD: 331 ± 48 imp/s; PD: 332 ± 49 imp/s; TM: 305 ± 37 imp/s; TriLo: 170 ± 25 imp/s; TriLa: 243 ± 38 imp/s; BicL: 171 ± 49 imp/s; BicS: 177 ± 63 imp/s; Br: 304 ± 93 imp/s; FCR: 129 ± 42 imp/s; FCU: 139 ± 40 imp/s; ECR: 142 ± 35 imp/s with SD across participants). However, the maximal simulated firing rates increased linearly with the increases in model coefficients (data not shown). Therefore, the conclusions drawn based on the data simulated at extremes using models with large coefficients will apply to the data obtained using models with lower coefficients.

**Figure 8:**
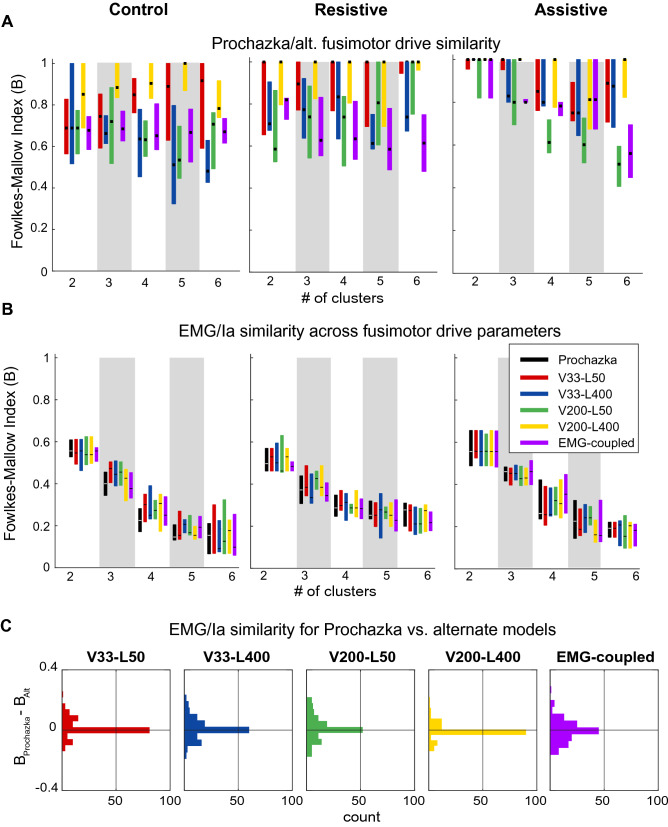
Fusimotor-based changes in the clustering of Ia profiles. (**A**) Similarity indices B between clusters produced by models with altered fusimotor coefficients and those produced by the original Prochazka model, boxes define the interquartile range across participants with the medians denoted by dots. (**B**) Similarity indices B between EMG and Ia clusters for models with altered coefficients. Formatting is the same as in (**A**). (**C**) The histograms of differences between B of the Prochazka model and each of the alternative Ia models across individual participants, tasks, and cluster subdivisions. Zero difference is denoted by horizontal lines.

We first evaluated how different static sets and α-γ coactivation can change the clustering structure of Ia profiles relative to that produced by the model with original coefficients (Prochazka model). We found that altering the Ia model coefficients did affect the clustering pattern of Ia signals as evident from the similarity indices between alternative models and the Prochazka model being less than 1 (Fig. 8A). The largest changes in Ia cluster structure were caused by the EMG-coupled Ia model simulating α-γ coactivation compared to those simulating altered static set. However, these changes in cluster structure did not increase the similarity between EMG and Ia profiles (Fig. 8B). Therefore, the last set of hypotheses stating that the similarity between Ia and EMG clusters is not increased by alternative fusimotor drives were not supported (Fig. 8C; Supplementary Tables S7–S11). This shows that the changes in the Ia afferent activity caused by known fusimotor input, such as static set or α-γ coactivation, are not likely to potentiate sufficiently the contribution of monosynaptic Ia feedback to the co-contraction of antagonists.

## Discussion

Here we addressed the question of the degree to which the Ia afferent activity from muscle spindles in antagonists can contribute to their co-contraction through monosynaptic feedback under normal conditions, such as reaching movements. We asked human participants to reach toward virtual targets at different locations, which instructed planar movements in a sagittal vertical plane. These reaching tasks were accompanied by different roles of passive limb dynamics, assistive or resistive. We found that EMG patterns changed between tasks and were associated with different levels of co-contraction, while the Ia patterns did not change between tasks and were primarily reciprocal between antagonists. Altering Ia model coefficients to simulate different types of fusimotor drive, such as static set and α-γ coactivation, did not change these conclusions. Although these results cannot rule out any given motor control theory, they do constrain the space of possible neural control solutions. Our results suggest a limited contribution of direct projections from the Ia afferents to muscle co-contraction, even with “simple” task-dependent changes in the fusimotor drive, such as static set and α-γ coactivation.

The reaching tasks selected for this study represent unique dynamical contexts experienced by the multisegmented limb during movement in the presence of gravity. This was reflected in different active muscle torques and mechanical muscle work around the major joints in the three tasks (Table 1). Motion in the Control and Assistive tasks was produced with reliance on passive interaction torques and gravity. In the Assistive task, this was accompanied by the coactivation of the largest group of muscles (Figs. 3, 4). This may have served to increase distal limb stiffness, which helped to stabilize the movement against the potentially de-stabilizing whiplash interactions between joints^11,40,50–53^. In contrast, motion in the Resistive task was produced against the opposing action of gravity and interaction torques between shoulder and elbow. This was accomplished with concentric contractions of two different groups of proximal and distal muscles, biarticular biceps and triceps muscles changed their coactivation patterns the most (Figs. 3, 4). Overall, our results suggest that the dynamical demands of each task define specific patterns of coactivation of agonist and antagonist muscles that form broadly defined proximal and distal groups. These flexible task-dependent groups of coactivating muscles may reflect the neural compensation of limb dynamics through limb impedance^12–14,50,51,54,55^.

There is a known monosynaptic relationship between Ia afferents and motoneurons innervating the same and synergistic muscles that underlies stretch reflexes, which compensate for perturbations. This anatomical arrangement with high gain, i.e. strong coupling, could result in similar profiles of the activity of Ia afferents and the profiles of the activity of homologous motoneurons, measured with EMG. Here, we tested this idea using two methods, hierarchal clustering of the correlation matrix between simulated Ia and EMG profiles and shared variance between profiles of antagonist co-contraction and Ia profiles. Hierarchal clustering revealed low similarity between Ia and EMG clusters, that resulted from inconsistent positive correlations between EMG and Ia profiles from the same muscles across tasks (Fig. 7; Supplementary Table S5). The comparison of co-contraction profiles with Ia profiles showed that only one of the antagonistic muscles was associated with positively correlated profiles, but not the other, and those relationships varied across tasks (Supplementary Table S6). Furthermore, we found that changing the parameters of the Ia model to simulate different levels of fusimotor static set between tasks did not increase the similarity between Ia and EMG clusters (Fig. 8). This suggests that simply changing the constant level of fusimotor drive between tasks cannot transform the Ia afferent activity so that it could contribute more to the co-contraction of antagonistic muscles observed during reaching movements. This may explain the findings of decreased gain of H-reflexes, which are indicative of the strength of the monosynaptic connection between the Ia afferents and α motoneurons, during tasks that require more co-contraction. For example, learning to co-contract antagonistic muscles during standing reduces the gain of soleus H-reflex in humans^56^. A recent simulation study has also shown that low gain of afferent feedback, both Ia and Ib, combined with co-contraction driven by mainly descending signals results in the optimal combination of stable control of movement and timely response to perturbations^57^. Altogether, this suggests that the monosynaptic Ia afferent feedback needs to be modulated nonlinearly during movement to contribute significantly to the co-contraction of antagonistic muscles.

Here we explored one type of nonlinear fusimotor drive that coupled the changes in muscle spindle sensitivity to muscle length and its rate of change to the activity of homonymous motoneurons. Such α-γ coactivation is thought to potentiate the recruitment of homonymous motor pools, increase muscle stiffness, and decrease the response times to perturbation^58,59^. Our results have shown that the nonlinear transformation of the Ia afferent signal by α-γ coactivation can change the profiles and, consequently, the clustering of Ia afferent signals more than all other models (Fig. 8A, EMG-coupled model). However, these changes were not enough to alter the Ia afferent profiles in a way that would reflect muscle co-contraction (Fig. 8B, C). This suggests that another type of nonlinear transformation of Ia afferent signals that is independent of signals modulating the activity of α motoneurons is required for Ia afferent-based co-contraction of antagonistic muscles. This transformation could either be applied through a complex nonlinear profile of fusimotor drive or nonlinear modulation of the gain of Ia afferent feedback onto the α motoneurons through presynaptic inhibition and/or spinal interneurons. Our results and approach can be used to test the first possibility. We can derive the temporal profile of the static and dynamic γ motoneuronal activity that would create the task-dependent coactivation pattern seen in EMG by solving Eq. (2) with least squares for two separate *a* coefficients (static and dynamic) for every phase of movement using EMG co-contraction as a cost function. The second possibility is more open-ended. The second type of nonlinear transformation may be accomplished by state-dependent nonlinear presynaptic inhibition of the monosynaptic pathway from Ia afferents to α motoneurons in some of the co-contracting antagonistic muscles^56,60^ or by state-dependent nonlinear inhibition of Ia interneurons that mediate the reciprocal inhibition of antagonists (for review see^61^). The propriospinal system can also be engaged in task-dependent modulation of afferent feedback gains^62^. Ultimately, the task-dependency is thought to be determined by the higher-level neural circuits that modulate presynaptic inhibition, fusimotor drive, and the activity of spinal interneurons^29,58,59,62,63^. Future perturbation studies that alter the Ia feedback, for example with vibration, during reaching with different dynamical contexts is the next logical step to test the predictions from this computational study.

A potential limitation of the Ia afferent model used here is its simplicity. More complex models of Ia afferents take into account intrafusal muscle properties and may have somewhat different profiles of Ia afferent activity^64,65^. Specifically, these models capture the transient bursts in afferent firing due to short range stiffness of the intrafusal muscle fiber. It is not known how these transient bursts are used by the nervous system, a recent paper suggested they may help sense changes in muscle force^66^. However, the scientific consensus is that the muscle spindle is primarily a sensor of muscle length and its rate of change, so that all models capture these features in their predictions of Ia afferent discharge. Therefore, our conclusions from the simple model are likely to be generalizable to simulations with other more complex models.

Another limitation our Ia simulations is the assumption that the muscle rest lengths is a halfway length between min and max of all possible muscle lengths across the whole physiological range of motion simulated in Gritsenko et al.^1^. We observed that the distributions of muscle lengths across degrees of freedom in the Gritsenko et al.^1^ were often not normal. Therefore, the half-way estimate of rest length could bias it to be outside of the most common operational range. To mitigate this limitation and test the generality of our results, we re-ran all analyses using rest lengths calculated differently. The new rest lengths were calculated as median lengths using the distributions from Gritsenko et al.^1^. The results were the same (data no included), further supporting the generalizability of our results.

The linear correlative approach used here to compare the primary Ia and EMG signals does not take into account the non-linear aspects of the transformation between them through the motoneuron pool. However, Farina et al.^35^ have shown that motoneuron pools, unlike individual motoneurons, display linearizing properties in transforming the common synaptic input into the neural drive to the muscle, i.e. EMG. This shifts the bulk of non-linearities in the transformation from the monosynaptic Ia feedback to EMG toward other synaptic inputs, such as spinal interneuronal and descending inputs discussed above. Moreover, the non-linearities in the transformation from the activity of a motoneuron pool to EMG are likely to be minimal in the low range of ~ 5–10% of maximal voluntary contraction during reaching^38,39^ examined here. At that range, the rate coding of recruited motor units is likely to drive linearly the EMG amplitude and, thus, muscle force^36^. Therefore, within the constraints of our experiment the non-linear transformation of the monosynaptic primary Ia inputs to the motoneuron pool into EMG is likely to capture a smaller component of the transformation than the linear one quantified here.

## Supplementary information

Supplementary file1 (DOCX 71 kb)

## Data Availability

The datasets generated during and/or analyzed during the current study are available from the corresponding author on reasonable request.
