# Supplementary material for: Computational evidence for nonlinear feedforward modulation of fusimotor drive to antagonistic co-contracting muscles
Source: Sci Rep. 2020 Jun 30;10:10625. doi: 10.1038/s41598-020-67403-w (PMC7326973; doi:10.1038/s41598-020-67403-w)
Supplement: Supplementary file 1 — Supplementary file1 (DOCX 71 kb) [file 41598_2020_67403_MOESM1_ESM.docx]

**Computational evidence for nonlinear feedforward modulation of fusimotor drive to antagonistic co-contracting muscles**

Russell L. Hardesty, Matthew T. Boots, Sergiy Yakovenko, and Valeriya Gritsenko

**Supplementary Table S1: Shared variance between EMG profiles of antagonist pairs.**

|  | Control | Resistive | Assistive |
| --- | --- | --- | --- |
| **Pec/TM** | 0.23 ± 0.33 | 0.13 ± 0.33 | 0.2 ± 0.27 |
| **AD/PD** | 0.07 ± 0.36 | 0.22 ± 0.28 | -0.05 ± 0.35 |
| **BicL/TriLo** | 0.32 ± 0.29 | -0.06 ± 0.31 | 0.3 ± 0.3 |
| **BicS/TriLa** | 0.16 ± 0.16 | 0.07 ± 0.24 | 0.28 ± 0.21 |
| **BR/TriLa** | 0.25 ± 0.28 | 0.14 ± 0.25 | 0.34 ± 0.3 |
| **FCR/ECR** | 0.16 ± 0.35 | 0.17 ± 0.37 | 0.35 ± 0.33 |
| **FCU/ECR** | 0.12 ± 0.2 | 0.02 ± 0.24 | 0.39 ± 0.26 |

Variance explained (R^2^) values were calculated between antagonist EMG signals for each movement. The means ± standard deviations are displayed across participants. Negative R^2^ values refer to linear regressions in which the slope was negative (inverse relationship). **The amount of co-contraction changes between tasks.**

**Supplementary Table S2: Shared variance between Ia profiles of antagonist pairs.**

|  | Control | Resistive | Assistive |
| --- | --- | --- | --- |
| **Pec/TM** | 0.19 ± 0.41 | 0.5 ± 0.56 | 0.4 ± 0.45 |
| **AD/PD** | -0.98 ± 0.01 | -0.96 ± 0.02 | -0.98 ± 0.02 |
| **BicL/TriLo** | -0.56 ± 0.14 | -0.58 ± 0.25 | -0.84 ± 0.22 |
| **BicS/TriLa** | -0.97 ± 0.03 | -0.89 ± 0.13 | -0.95 ± 0.06 |
| **BR/TriLa** | -0.75 ± 0.23 | -0.96 ± 0.05 | -0.75 ± 0.11 |
| **FCR/ECR** | -0.33 ± 0.45 | -0.03 ± 0.34 | -0.51 ± 0.36 |
| **FCU/ECR** | -0.52 ± 0.33 | -0.17 ± 0.3 | -0.61 ± 0.34 |

Variance explained (R^2^) values were calculated between antagonist Ia signals for each movement. The means ± standard deviations are displayed across participants. Negative R^2^ values refer to linear regressions in which the slope was negative (inverse relationship). **Most antagonist pairs show strong inverse correlations reflecting the reciprocal feedback from Ia afferents.**

**Supplementary Table S3: The similarity in cluster structure between Ia and Muscle Length profiles.**

| **Control** | | | | | |
| --- | --- | --- | --- | --- | --- |
| Subject | Cluster 2 | Cluster 3 | Cluster 4 | Cluster 5 | Cluster 6 |
| 1 | 0.15 | 0.09 | 0.14 | 0.12 | 0.05 |
| 2 | 0.24 | 0.13 | 0.02 | 0.06 | 0.01 |
| 3 | 0.22 | 0.30 | 0.17 | 0.11 | 0.12 |
| 4 | 0.48 | 0.14 | 0.02 | 0.05 | 0.05 |
| 5 | 0.57 | 0.05 | 0.01 | 0.01 | **<0.01** |
| 6 | 0.23 | 0.30 | 0.18 | 0.27 | 0.15 |
| 7 | 0.58 | 0.28 | 0.31 | 0.06 | 0.02 |
| 8 | 0.08 | 0.03 | 0.02 | 0.05 | **<0.01** |
| 9 | 0.08 | 0.08 | 0.02 | 0.25 | 0.10 |
| Combined | 0.09 | **<0.01** | **<0.01** | **<0.01** | **<0.01** |
|  |  |  |  |  |  |
| **Resistive** | | | | | |
| Subject | Cluster 2 | Cluster 3 | Cluster 4 | Cluster 5 | Cluster 6 |
| 1 | 0.04 | 0.02 | 0.01 | 0.01 | **<0.01** |
| 2 | **<0.01** | **<0.01** | **<0.01** | **<0.01** | **<0.01** |
| 3 | 0.17 | 0.04 | **<0.01** | **<0.01** | **<0.01** |
| 4 | 0.23 | 0.32 | 0.09 | 0.13 | **<0.01** |
| 5 | 0.01 | 0.07 | 0.10 | 0.08 | **<0.01** |
| 6 | **<0.01** | **<0.01** | 0.01 | **<0.01** | **<0.01** |
| 7 | **<0.01** | 0.34 | 0.28 | **<0.01** | **<0.01** |
| 8 | 0.02 | 0.09 | 0.03 | 0.02 | 0.01 |
| 9 | 0.58 | 0.12 | 0.03 | 0.02 | 0.01 |
| Combined | **<0.01** | **<0.01** | **<0.01** | **<0.01** | **<0.01** |
|  |  |  |  |  |  |
| **Assistive** | | | | | |
| Subject | Cluster 2 | Cluster 3 | Cluster 4 | Cluster 5 | Cluster 6 |
| 1 | 0.22 | 0.08 | 0.04 | 0.12 | 0.04 |
| 2 | 0.06 | 0.28 | 0.02 | 0.04 | **<0.01** |
| 3 | 0.07 | 0.28 | 0.10 | 0.45 | 0.11 |
| 4 | 0.09 | 0.26 | 0.12 | 0.18 | 0.01 |
| 5 | 0.09 | 0.08 | 0.06 | 0.45 | 0.10 |
| 6 | 0.02 | 0.03 | 0.08 | 0.11 | 0.04 |
| 7 | 0.09 | 0.29 | 0.14 | 0.18 | 0.15 |
| 8 | 0.01 | 0.28 | 0.16 | 0.43 | 0.42 |
| 9 | 0.01 | 0.15 | 0.15 | 0.10 | 0.08 |
| Combined | **<0.01** | 0.01 | **<0.01** | 0.03 | **<0.01** |

P-values are the probability of falsely rejecting the null hypothesis that the similarity index B between Ia and muscle length clusters is equal to that of permuted clusters. The combined p-values across cluster subdivisions were obtained using the Fisher's combined probability test (Fisher, 1970). The significant alpha was set to 0.0056 to adjust for repeating tests across participants. **The significant similarity index at multiple cluster subdivisions confirms that the same muscles that shorten or lengthen together also have similar Ia feedback across multiple postures or movements.**

**Supplementary Table S4: Shared variance between Ia and EMG from homonymous muscles.**

|  | Control | Resistive | Assistive |
| --- | --- | --- | --- |
| **Pec** | -0.33 ± 0.31 | -0.03 ± 0.3 | -0.29 ± 0.35 |
| **AD** | 0.13 ± 0.57 | -0.25 ± 0.4 | -0.04 ± 0.46 |
| **PD** | -0.11 ± 0.25 | 0.15 ± 0.46 | -0.11 ± 0.42 |
| **TM** | -0.04 ± 0.39 | 0.15 ± 0.37 | -0.08 ± 0.5 |
| **TriLo** | 0.06 ± 0.4 | 0.20 ± 0.25 | 0.23 ± 0.18 |
| **TriLa** | 0.52 ± 0.27 | -0.01 ± 0.29 | 0.31 ± 0.36 |
| **BicL** | -0.61 ± 0.21 | 0.07 ± 0.11 | -0.08 ± 0.29 |
| **BicS** | -0.3 ± 0.24 | -0.28 ± 0.34 | -0.27 ± 0.3 |
| **BR** | -0.25 ± 0.3 | 0.03 ± 0.07 | -0.05 ± 0.47 |
| **FCR** | -0.02 ± 0.31 | -0.04 ± 0.27 | 0.18 ± 0.38 |
| **FCU** | 0.01 ± 0.19 | 0.07 ± 0.25 | 0.15 ± 0.31 |
| **ECR** | -0.13 ± 0.22 | -0.03 ± 0.15 | -0.07 ± 0.35 |

Variance explained (R^2^) values were calculated between homonymous EMG and Ia signals for each movement, e.g. BicL EMG with BicL Ia. The means ± standard deviations are displayed across participants. Negative R^2^ values refer to linear regressions in which the slope was negative (inverse relationship). **The large number of negative correlations indicate primarily reciprocal relationships between Ia afferent feedback and the activation of its parent muscle.**

**Supplementary Table S5: The similarity in cluster structure between Ia and EMG Profiles.**

| **Control** | | | | | |
| --- | --- | --- | --- | --- | --- |
| Subject | Cluster 2 | Cluster 3 | Cluster 4 | Cluster 5 | Cluster 6 |
| 1 | 0.59 | 0.45 | 0.40 | 0.44 | 0.56 |
| 2 | **<0.01** | 0.23 | 0.71 | 0.90 | 0.27 |
| 3 | 0.09 | 0.42 | 0.70 | 0.69 | 0.90 |
| 4 | 0.17 | 0.30 | 0.16 | 0.07 | 0.03 |
| 5 | **<0.01** | 0.29 | 0.76 | 0.52 | 0.48 |
| 6 | 0.44 | 0.12 | 0.86 | 0.81 | 0.61 |
| 7 | 0.04 | 0.48 | 0.23 | 0.53 | 0.41 |
| 8 | 0.06 | 0.37 | 0.92 | 0.69 | 0.83 |
| 9 | 0.43 | 0.17 | 0.57 | 0.16 | 0.06 |
| Combined | **<0.01** | 0.21 | 0.85 | 0.64 | 0.27 |
|  |  |  |  |  |  |
| **Resistive** | | | | | |
| Subject | Cluster 2 | Cluster 3 | Cluster 4 | Cluster 5 | Cluster 6 |
| 1 | 0.19 | 0.40 | 0.20 | 0.12 | 0.03 |
| 2 | 0.23 | 0.42 | 0.26 | 0.15 | 0.08 |
| 3 | 0.10 | 0.18 | 0.54 | 0.26 | 0.08 |
| 4 | 0.01 | 0.09 | 0.04 | 0.08 | 0.09 |
| 5 | **<0.01** | 0.31 | 0.73 | 0.08 | 0.04 |
| 6 | 0.22 | 0.40 | 0.17 | 0.31 | 0.66 |
| 7 | 0.06 | 0.73 | 0.53 | 0.52 | 0.37 |
| 8 | 0.52 | 0.71 | 0.39 | 0.12 | 0.04 |
| 9 | 0.25 | 0.01 | 0.09 | 0.14 | 0.12 |
| Combined | **<0.01** | 0.09 | 0.11 | 0.02 | **<0.01** |
|  |  |  |  |  |  |
| **Assistive** | | | | | |
| Subject | Cluster 2 | Cluster 3 | Cluster 4 | Cluster 5 | Cluster 6 |
| 1 | **<0.01** | 0.39 | 0.74 | 0.17 | 0.19 |
| 2 | 0.23 | 0.02 | 0.01 | 0.05 | 0.02 |
| 3 | 0.01 | 0.07 | 0.52 | 0.20 | 0.15 |
| 4 | **<0.01** | 0.48 | 0.32 | 0.49 | 0.12 |
| 5 | **<0.01** | 0.15 | 0.13 | 0.28 | 0.27 |
| 6 | 0.18 | 0.26 | 0.03 | 0.13 | 0.26 |
| 7 | 0.16 | 0.30 | 0.93 | 0.82 | 0.32 |
| 8 | 0.55 | 0.76 | 0.90 | 0.43 | 0.57 |
| 9 | 0.15 | 0.29 | 0.46 | 0.83 | 0.41 |
| Combined | **<0.01** | 0.06 | 0.09 | 0.18 | 0.04 |

P-values are the probability of falsely rejecting the null hypothesis that the similarity index B between Ia and EMG clusters is equal to that of permuted clusters. The combined p-values across cluster subdivisions were obtained using the Fisher's combined probability test (Fisher, 1970). The significant alpha was set to 0.0056 to adjust for repeating tests across participants. **The significant similarity index at 2-cluster subdivision indicates that mostly the same muscles group into the 2 main categories of flexors and extensors based on EMG and Ia profile. The insignificant similarity index indicates spurious match between clusters.**

**Supplementary Table S6: Shared variance between co-contraction and Ia profiles.**

| **Control** | | | | | |
| --- | --- | --- | --- | --- | --- |
|  | Pec_TM | AD_PD | BicL_TriLo | BicS_TriLa | FCR_ECR |
| **Pec** | **0.11 ± 0.26** | 0.14 ± 0.5 | -0.21 ± 0.4 | -0.25 ± 0.44 | -0.13 ± 0.51 |
| **AD** | 0.05 ± 0.37 | **-0.27 ± 0.48** | 0.34 ± 0.41 | 0.41 ± 0.4 | 0.34 ± 0.49 |
| **PD** | -0.07 ± 0.39 | **0.3 ± 0.46** | -0.35 ± 0.36 | -0.38 ± 0.39 | -0.34 ± 0.5 |
| **TM** | **0.01 ± 0.33** | 0.36 ± 0.4 | -0.34 ± 0.35 | -0.32 ± 0.42 | -0.28 ± 0.53 |
| **TriLo** | -0.05 ± 0.37 | 0.2 ± 0.32 | **-0.27 ± 0.26** | -0.17 ± 0.26 | -0.23 ± 0.43 |
| **TriLa** | -0.27 ± 0.31 | -0.1 ± 0.18 | -0.02 ± 0.29 | **0.14 ± 0.26** | 0.03 ± 0.14 |
| **BicL** | 0.16 ± 0.38 | -0.02 ± 0.2 | **0.1 ± 0.17** | -0.02 ± 0.22 | 0.04 ± 0.19 |
| **BicS** | 0.26 ± 0.29 | 0.15 ± 0.23 | -0.07 ± 0.36 | **-0.21 ± 0.27** | -0.06 ± 0.17 |
| **BR** | 0.27 ± 0.29 | 0.07 ± 0.19 | 0.04 ± 0.25 | -0.1 ± 0.22 | 0.01 ± 0.17 |
| **FCR** | -0.1 ± 0.3 | 0.01 ± 0.34 | -0.12 ± 0.31 | -0.1 ± 0.21 | **-0.1 ± 0.24** |
| **FCU** | -0.07 ± 0.3 | -0.01 ± 0.31 | -0.07 ± 0.29 | -0.08 ± 0.22 | -0.06 ± 0.24 |
| **ECR** | -0.09 ± 0.3 | 0.04 ± 0.31 | -0.05 ± 0.25 | -0.04 ± 0.13 | **-0.1 ± 0.19** |
|  |  |  |  |  |  |
| **Resistive** | | | | | |
|  | Pec_TM | AD_PD | BicL_TriLo | BicS_TriLa | FCR_ECR |
| **Pec** | **0.11 ± 0.26** | 0.14 ± 0.5 | -0.21 ± 0.4 | -0.25 ± 0.44 | -0.13 ± 0.51 |
| **AD** | 0.06 ± 0.37 | **-0.28 ± 0.48** | 0.35 ± 0.4 | 0.41 ± 0.4 | 0.34 ± 0.49 |
| **PD** | -0.07 ± 0.41 | **0.3 ± 0.44** | -0.34 ± 0.36 | -0.36 ± 0.39 | -0.33 ± 0.49 |
| **TM** | **0.01 ± 0.33** | 0.36 ± 0.4 | -0.34 ± 0.35 | -0.32 ± 0.42 | -0.28 ± 0.53 |
| **TriLo** | -0.05 ± 0.37 | 0.18 ± 0.3 | **-0.23 ± 0.24** | -0.14 ± 0.24 | -0.2 ± 0.4 |
| **TriLa** | -0.27 ± 0.31 | -0.1 ± 0.18 | -0.02 ± 0.29 | **0.14 ± 0.26** | 0.03 ± 0.14 |
| **BicL** | 0.16 ± 0.38 | -0.02 ± 0.2 | **0.1 ± 0.17** | -0.02 ± 0.22 | 0.04 ± 0.19 |
| **BicS** | 0.26 ± 0.29 | 0.15 ± 0.23 | -0.07 ± 0.36 | **-0.21 ± 0.27** | -0.06 ± 0.17 |
| **BR** | 0.27 ± 0.29 | 0.07 ± 0.19 | 0.04 ± 0.25 | -0.1 ± 0.22 | 0 ± 0.17 |
| **FCR** | -0.1 ± 0.3 | 0.01 ± 0.34 | -0.12 ± 0.31 | -0.1 ± 0.21 | **-0.1 ± 0.24** |
| **FCU** | -0.07 ± 0.3 | -0.01 ± 0.31 | -0.07 ± 0.29 | -0.08 ± 0.22 | -0.06 ± 0.24 |
| **ECR** | -0.09 ± 0.3 | 0.04 ± 0.31 | -0.05 ± 0.25 | -0.04 ± 0.13 | **-0.1 ± 0.19** |
|  |  |  |  |  |  |
| **Assistive** | | | | | |
|  | Pec_TM | AD_PD | BicL_TriLo | BicS_TriLa | FCR_ECR |
| **Pec** | **-0.26 ± 0.54** | -0.14 ± 0.25 | -0.15 ± 0.4 | -0.28 ± 0.42 | -0.34 ± 0.38 |
| **AD** | 0.21 ± 0.45 | **0.01 ± 0.46** | 0.26 ± 0.39 | 0.44 ± 0.42 | 0.41 ± 0.53 |
| **PD** | -0.24 ± 0.45 | **-0.01 ± 0.43** | -0.27 ± 0.35 | -0.48 ± 0.39 | -0.42 ± 0.52 |
| **TM** | **-0.17 ± 0.42** | 0.03 ± 0.42 | -0.24 ± 0.32 | -0.45 ± 0.4 | -0.5 ± 0.49 |
| **TriLo** | 0.22 ± 0.39 | 0.1 ± 0.28 | **0.24 ± 0.2** | 0.24 ± 0.22 | 0.36 ± 0.41 |
| **TriLa** | 0.18 ± 0.43 | 0.04 ± 0.42 | 0.24 ± 0.34 | **0.32 ± 0.35** | 0.41 ± 0.51 |
| **BicL** | -0.19 ± 0.44 | -0.09 ± 0.37 | **-0.24 ± 0.3** | -0.26 ± 0.29 | -0.4 ± 0.47 |
| **BicS** | -0.16 ± 0.44 | -0.06 ± 0.37 | -0.24 ± 0.3 | **-0.27 ± 0.31** | -0.41 ± 0.47 |
| **BR** | -0.04 ± 0.41 | 0.15 ± 0.45 | -0.14 ± 0.43 | -0.32 ± 0.49 | -0.34 ± 0.55 |
| **FCR** | -0.06 ± 0.39 | 0.02 ± 0.3 | 0.06 ± 0.28 | 0.2 ± 0.33 | **0.19 ± 0.42** |
| **FCU** | -0.05 ± 0.46 | -0.03 ± 0.36 | 0.07 ± 0.27 | 0.24 ± 0.33 | 0.22 ± 0.38 |
| **ECR** | 0.1 ± 0.5 | 0.03 ± 0.28 | -0.04 ± 0.29 | -0.17 ± 0.41 | **-0.06 ± 0.32** |

Variance explained (R^2^) values were calculated between EMG co-contraction (columns) and Ia signals (rows) for each movement. Bolded values are for muscles comprising the co-contraction pairs. The means ± standard deviations are displayed across participants. Negative R^2^ values refer to linear regressions in which the slope was negative (inverse relationship). **For** **most antagonist pairs, with the exception of Pec_TM, the co-contraction was negatively correlated with one or both Ia signals from the corresponding muscles.**

**Supplementary Table S7: The similarity in cluster structure between Ia and EMG Profiles for V33-L50 model.**

| **Control** | | | | | |
| --- | --- | --- | --- | --- | --- |
| Subject | Cluster 2 | Cluster 3 | Cluster 4 | Cluster 5 | Cluster 6 |
| 1 | 0.54 | 0.17 | 0.36 | 0.44 | 0.54 |
| 2 | 0.34 | 0.23 | 0.70 | 0.92 | 0.24 |
| 3 | 0.44 | 0.22 | 0.15 | 0.47 | 0.90 |
| 4 | 0.06 | 0.05 | 0.15 | 0.08 | 0.02 |
| 5 | **<0.01** | 0.24 | 0.56 | 0.51 | 0.51 |
| 6 | 0.12 | 0.51 | 0.74 | 0.78 | 0.57 |
| 7 | 0.05 | 0.11 | 0.03 | 0.03 | 0.04 |
| 8 | 0.56 | 0.76 | 0.78 | 0.64 | 0.81 |
| 9 | 0.33 | 0.20 | 0.39 | 0.35 | 0.05 |
| Combined | **<0.01** | 0.06 | 0.25 | 0.34 | 0.07 |
|  |  |  |  |  |  |
| **Resistive** | | | | | |
| Subject | Cluster 2 | Cluster 3 | Cluster 4 | Cluster 5 | Cluster 6 |
| 1 | 0.01 | 0.25 | 0.22 | 0.06 | 0.03 |
| 2 | 0.22 | 0.42 | 0.26 | 0.11 | 0.08 |
| 3 | 0.09 | 0.18 | 0.20 | 0.26 | 0.07 |
| 4 | 0.02 | 0.02 | 0.04 | 0.07 | 0.09 |
| 5 | **<0.01** | 0.36 | 0.69 | 0.08 | 0.06 |
| 6 | 0.23 | 0.15 | 0.02 | 0.31 | 0.23 |
| 7 | 0.08 | 0.71 | 0.51 | 0.50 | 0.35 |
| 8 | 0.24 | 0.46 | 0.81 | 0.45 | 0.28 |
| 9 | 0.24 | **<0.01** | 0.09 | 0.13 | 0.13 |
| Combined | **<0.01** | **<0.01** | 0.03 | 0.02 | **<0.01** |
|  |  |  |  |  |  |
| **Assistive** | | | | | |
| Subject | Cluster 2 | Cluster 3 | Cluster 4 | Cluster 5 | Cluster 6 |
| 1 | **<0.01** | 0.32 | 0.78 | 0.56 | 0.21 |
| 2 | 0.22 | 0.02 | 0.01 | 0.03 | 0.03 |
| 3 | 0.02 | 0.08 | 0.68 | 0.53 | 0.26 |
| 4 | **<0.01** | 0.49 | 0.10 | 0.41 | 0.12 |
| 5 | **<0.01** | 0.48 | 0.32 | 0.47 | 0.23 |
| 6 | 0.02 | 0.12 | 0.04 | 0.18 | 0.24 |
| 7 | 0.16 | 0.32 | 0.93 | 0.56 | 0.38 |
| 8 | 0.55 | 0.77 | 0.49 | 0.33 | 0.13 |
| 9 | 0.04 | 0.31 | 0.48 | 0.37 | 0.43 |
| Combined | **<0.01** | 0.07 | 0.08 | 0.24 | 0.03 |

The data is presented as in Supplementary Table S5. **Lowering the Ia afferent sensitivity to both muscle length and its rate of change did not increase the match between EMG and Ia clusters.**

**Supplementary Table S8: The similarity in cluster structure between Ia and EMG Profiles for V33-L400 model.**

| **Control** | | | | | |
| --- | --- | --- | --- | --- | --- |
| Subject | Cluster 2 | Cluster 3 | Cluster 4 | Cluster 5 | Cluster 6 |
| 1 | 0.54 | 0.17 | 0.36 | 0.44 | 0.54 |
| 2 | 0.34 | 0.23 | 0.70 | 0.92 | 0.24 |
| 3 | 0.44 | 0.22 | 0.15 | 0.47 | 0.90 |
| 4 | 0.06 | 0.05 | 0.15 | 0.08 | 0.02 |
| 5 | **<0.01** | 0.24 | 0.56 | 0.51 | 0.51 |
| 6 | 0.12 | 0.51 | 0.74 | 0.78 | 0.57 |
| 7 | 0.05 | 0.11 | 0.03 | 0.03 | 0.04 |
| 8 | 0.56 | 0.76 | 0.78 | 0.64 | 0.81 |
| 9 | 0.33 | 0.20 | 0.39 | 0.35 | 0.05 |
| Combined | **<0.01** | 0.06 | 0.25 | 0.34 | 0.07 |
|  |  |  |  |  |  |
| **Resistive** | | | | | |
| Subject | Cluster 2 | Cluster 3 | Cluster 4 | Cluster 5 | Cluster 6 |
| 1 | 0.07 | 0.33 | 0.69 | 0.54 | 0.41 |
| 2 | 0.24 | 0.52 | 0.11 | 0.03 | 0.04 |
| 3 | 0.25 | 0.40 | 0.78 | 0.75 | 0.42 |
| 4 | 0.01 | 0.10 | 0.06 | 0.08 | 0.09 |
| 5 | 0.36 | 0.41 | 0.71 | 0.03 | 0.05 |
| 6 | 0.58 | 0.83 | 0.26 | 0.37 | 0.42 |
| 7 | 0.57 | 0.45 | 0.71 | 0.90 | 0.37 |
| 8 | **<0.01** | 0.11 | 0.04 | 0.04 | 0.03 |
| 9 | 0.21 | 0.01 | 0.08 | 0.14 | 0.12 |
| Combined | **<0.01** | 0.08 | 0.08 | 0.01 | **<0.01** |
|  |  |  |  |  |  |
| **Assistive** | | | | | |
| Subject | Cluster 2 | Cluster 3 | Cluster 4 | Cluster 5 | Cluster 6 |
| 1 | **<0.01** | 0.43 | 0.80 | 0.58 | 0.20 |
| 2 | 0.23 | 0.02 | 0.01 | 0.04 | 0.08 |
| 3 | 0.02 | 0.08 | 0.54 | 0.47 | 0.37 |
| 4 | **<0.01** | 0.49 | 0.16 | 0.16 | 0.14 |
| 5 | **<0.01** | 0.50 | 0.36 | 0.42 | 0.28 |
| 6 | 0.17 | 0.11 | **<0.01** | 0.17 | 0.39 |
| 7 | 0.13 | 0.30 | 0.53 | 0.22 | 0.19 |
| 8 | 0.56 | 0.75 | 0.71 | 0.43 | 0.53 |
| 9 | 0.18 | 0.48 | 0.48 | 0.26 | 0.34 |
| Combined | **<0.01** | 0.09 | **<0.01** | 0.11 | 0.12 |

The data is presented as in Supplementary Table S5. **Lowering the Ia afferent sensitivity to muscle length while increasing its sensitivity to the rate of change in muscle length did not increase the match between EMG and Ia clusters.**

**Supplementary Table S9: The similarity in cluster structure between Ia and EMG Profiles for V200-L50.**

| **Control** | | | | | |
| --- | --- | --- | --- | --- | --- |
| Subject | Cluster 2 | Cluster 3 | Cluster 4 | Cluster 5 | Cluster 6 |
| 1 | 0.56 | 0.48 | 0.58 | 0.79 | 0.54 |
| 2 | 0.34 | 0.22 | 0.35 | 0.30 | 0.24 |
| 3 | 0.44 | 0.21 | 0.29 | 0.37 | 0.90 |
| 4 | 0.06 | 0.07 | 0.13 | **<0.01** | **<0.01** |
| 5 | **<0.01** | 0.24 | 0.66 | 0.90 | 0.73 |
| 6 | 0.09 | 0.48 | 0.75 | 0.49 | 0.65 |
| 7 | 0.16 | 0.03 | 0.03 | 0.20 | 0.13 |
| 8 | 0.53 | 0.19 | 0.77 | 0.34 | 0.78 |
| 9 | 0.32 | 0.24 | 0.41 | 0.21 | 0.04 |
| Combined | **<0.01** | 0.04 | 0.29 | 0.07 | 0.03 |
|  |  |  |  |  |  |
| **Resistive** | | | | | |
| Subject | Cluster 2 | Cluster 3 | Cluster 4 | Cluster 5 | Cluster 6 |
| 1 | 0.02 | 0.23 | 0.22 | 0.14 | 0.03 |
| 2 | 0.23 | 0.16 | 0.36 | 0.11 | 0.08 |
| 3 | 0.55 | 0.21 | 0.54 | 0.26 | 0.07 |
| 4 | **<0.01** | 0.03 | 0.06 | 0.08 | 0.08 |
| 5 | **<0.01** | 0.31 | 0.71 | 0.53 | 0.26 |
| 6 | 0.57 | 0.77 | 0.36 | 0.10 | 0.53 |
| 7 | **<0.01** | 0.09 | 0.33 | 0.18 | 0.34 |
| 8 | 0.22 | 0.48 | 0.83 | 0.62 | 0.31 |
| 9 | 0.21 | 0.23 | 0.34 | 0.10 | 0.11 |
| Combined | **<0.01** | 0.05 | 0.36 | 0.03 | **<0.01** |
|  |  |  |  |  |  |
| **Assistive** | | | | | |
| Subject | Cluster 2 | Cluster 3 | Cluster 4 | Cluster 5 | Cluster 6 |
| 1 | **<0.01** | 0.35 | 0.43 | 0.54 | 0.53 |
| 2 | 0.22 | 0.02 | 0.01 | 0.06 | 0.05 |
| 3 | 0.01 | 0.09 | 0.03 | 0.17 | 0.24 |
| 4 | **<0.01** | 0.46 | 0.11 | 0.18 | 0.03 |
| 5 | **<0.01** | 0.18 | 0.37 | 0.40 | 0.43 |
| 6 | 0.02 | 0.27 | 0.63 | 0.41 | 0.23 |
| 7 | 0.15 | 0.33 | 0.53 | 0.23 | 0.57 |
| 8 | 0.25 | 0.62 | 0.23 | 0.06 | 0.09 |
| 9 | 0.17 | 0.49 | 0.48 | 0.90 | 0.43 |
| Combined | **<0.01** | 0.09 | 0.02 | 0.09 | 0.04 |

The data is presented as in Supplementary Table S5. **Increasing the Ia afferent sensitivity to muscle length while lowering its sensitivity to the rate of change in muscle length did not increase the match between EMG and Ia clusters.**

**Supplementary Table S10: The similarity in cluster structure between Ia and EMG Profiles for V200-L400.**

| **Control** | | | | | |
| --- | --- | --- | --- | --- | --- |
| Subject | Cluster 2 | Cluster 3 | Cluster 4 | Cluster 5 | Cluster 6 |
| 1 | 0.55 | 0.72 | 0.34 | 0.46 | 0.54 |
| 2 | 0.34 | 0.21 | 0.72 | 0.91 | 0.23 |
| 3 | 0.44 | 0.23 | 0.14 | 0.38 | 0.90 |
| 4 | 0.06 | 0.10 | 0.14 | 0.09 | 0.01 |
| 5 | **<0.01** | 0.28 | 0.80 | 0.52 | 0.39 |
| 6 | 0.48 | 0.27 | 0.75 | 0.82 | 0.59 |
| 7 | 0.05 | 0.49 | 0.22 | 0.55 | 0.25 |
| 8 | 0.06 | 0.39 | 0.77 | 0.62 | 0.79 |
| 9 | 0.31 | 0.19 | 0.12 | 0.35 | 0.05 |
| Combined | **<0.01** | 0.19 | 0.36 | 0.70 | 0.09 |
|  |  |  |  |  |  |
| **Resistive** | | | | | |
| Subject | Cluster 2 | Cluster 3 | Cluster 4 | Cluster 5 | Cluster 6 |
| 1 | 0.02 | 0.26 | 0.24 | 0.04 | 0.03 |
| 2 | 0.21 | 0.43 | 0.27 | 0.13 | 0.09 |
| 3 | 0.11 | 0.18 | 0.54 | 0.27 | 0.06 |
| 4 | 0.01 | 0.03 | 0.04 | 0.07 | 0.08 |
| 5 | **<0.01** | 0.33 | 0.72 | 0.08 | 0.05 |
| 6 | 0.21 | 0.16 | 0.02 | 0.29 | 0.42 |
| 7 | 0.06 | 0.72 | 0.56 | 0.51 | 0.36 |
| 8 | 0.23 | 0.49 | 0.81 | 0.45 | 0.25 |
| 9 | 0.22 | 0.02 | 0.09 | 0.11 | 0.12 |
| Combined | **<0.01** | 0.02 | 0.06 | 0.02 | **<0.01** |
|  |  |  |  |  |  |
| **Assistive** | | | | | |
| Subject | Cluster 2 | Cluster 3 | Cluster 4 | Cluster 5 | Cluster 6 |
| 1 | **<0.01** | 0.34 | 0.27 | 0.45 | 0.13 |
| 2 | 0.22 | 0.02 | 0.02 | 0.04 | 0.03 |
| 3 | 0.01 | 0.07 | 0.05 | 0.40 | 0.15 |
| 4 | **<0.01** | 0.48 | 0.30 | 0.40 | 0.13 |
| 5 | **<0.01** | 0.52 | 0.38 | 0.29 | 0.21 |
| 6 | 0.17 | 0.25 | 0.04 | 0.40 | 0.60 |
| 7 | 0.16 | 0.28 | 0.93 | 0.82 | 0.31 |
| 8 | 0.56 | 0.77 | 0.88 | 0.77 | 0.51 |
| 9 | 0.18 | 0.30 | 0.45 | 0.81 | 0.40 |
| Combined | **<0.01** | 0.08 | 0.04 | 0.50 | 0.05 |

The data is presented as in Supplementary Table S5. **Increasing the Ia afferent sensitivity to both muscle length and its rate of change did not increase the match between EMG and Ia clusters.**

**Supplementary Table S11: The similarity in cluster structure between Ia and EMG Profiles for EMG-Coupled model.**

| **Control** | | | | | |
| --- | --- | --- | --- | --- | --- |
| Subject | Cluster 2 | Cluster 3 | Cluster 4 | Cluster 5 | Cluster 6 |
| 1 | 0.23 | 0.65 | 0.25 | 0.61 | 0.88 |
| 2 | 0.34 | 0.32 | 0.81 | 0.23 | 0.48 |
| 3 | 0.47 | 0.41 | 0.09 | 0.50 | 0.48 |
| 4 | 0.02 | **<0.01** | 0.08 | 0.05 | 0.01 |
| 5 | 0.58 | 0.56 | 0.34 | 0.52 | 0.32 |
| 6 | 0.14 | 0.36 | 0.76 | 0.68 | 0.75 |
| 7 | 0.25 | 0.56 | 0.88 | 0.43 | 0.34 |
| 8 | 0.53 | 0.66 | 0.94 | 0.53 | 0.83 |
| 9 | **<0.01** | 0.31 | 0.56 | 0.07 | 0.02 |
| Combined | **<0.01** | 0.15 | 0.51 | 0.24 | 0.09 |
|  |  |  |  |  |  |
| **Resistive** | | | | | |
| Subject | Cluster 2 | Cluster 3 | Cluster 4 | Cluster 5 | Cluster 6 |
| 1 | 0.57 | 0.67 | 0.15 | 0.06 | 0.07 |
| 2 | 0.05 | 0.33 | 0.27 | 0.15 | 0.26 |
| 3 | 0.57 | 0.34 | 0.53 | 0.34 | 0.13 |
| 4 | 0.08 | 0.61 | 0.31 | 0.29 | 0.21 |
| 5 | 0.44 | 0.32 | 0.72 | 0.56 | 0.15 |
| 6 | 0.56 | 0.46 | 0.35 | 0.05 | 0.09 |
| 7 | 0.25 | 0.90 | 0.87 | 0.39 | 0.25 |
| 8 | 0.54 | 0.78 | 0.91 | 0.64 | 0.32 |
| 9 | 0.05 | 0.04 | 0.26 | 0.68 | 0.27 |
| Combined | 0.10 | 0.53 | 0.59 | 0.13 | 0.02 |
|  |  |  |  |  |  |
| **Assistive** | | | | | |
| Subject | Cluster 2 | Cluster 3 | Cluster 4 | Cluster 5 | Cluster 6 |
| 1 | **<0.01** | 0.81 | 0.43 | 0.79 | 0.24 |
| 2 | 0.22 | 0.05 | **<0.01** | 0.06 | 0.09 |
| 3 | 0.02 | 0.02 | 0.04 | 0.39 | 0.34 |
| 4 | **<0.01** | 0.44 | 0.16 | 0.52 | 0.49 |
| 5 | **<0.01** | 0.03 | 0.13 | 0.29 | 0.06 |
| 6 | 0.53 | 0.10 | **<0.01** | 0.17 | 0.38 |
| 7 | 0.16 | 0.30 | 0.93 | 0.82 | 0.36 |
| 8 | 0.23 | 0.75 | 0.69 | 0.43 | 0.20 |
| 9 | 0.04 | 0.31 | 0.24 | 0.80 | 0.62 |
| Combined | **<0.01** | 0.02 | **<0.01** | 0.45 | 0.12 |

The data is presented as in Supplementary Table S5. **Modulating the Ia afferent sensitivity to muscle length and its rate of change did not increase the match between EMG and Ia clusters, with the exception of 4-cluster subdivision in 2 participants.**
